# Optimizing Optical Flow Cytometry for Cell Volume-Based Sorting and Analysis

**DOI:** 10.1371/journal.pone.0016053

**Published:** 2011-01-20

**Authors:** Amit Tzur, Jodene K. Moore, Paul Jorgensen, Howard M. Shapiro, Marc W. Kirschner

**Affiliations:** 1 Department of Systems Biology, Harvard Medical School, Boston, Massachusetts, United States of America; 2 The Mina and Everard Goodman Faculty of Life Sciences and Advanced Materials and Nanotechnology Institute, Bar-Ilan University, Ramat-Gan, Israel; 3 Donnelly Centre for Cellular and Biomolecular Research, University of Toronto, Toronto, Canada; 4 The Center for Microbial Cytometry and Howard M. Shapiro, West Newton, Massachusetts, United States of America; Virginia Tech, United States of America

## Abstract

Cell size is a defining characteristic central to cell function and ultimately to tissue architecture. The ability to sort cell subpopulations of different sizes would facilitate investigation at genomic and proteomic levels of mechanisms by which cells attain and maintain their size. Currently available cell sorters, however, cannot directly measure cell volume electronically, and it would therefore be desirable to know which of the optical measurements that can be made in such instruments provide the best estimate of volume. We investigated several different light scattering and fluorescence measurements in several different cell lines, sorting cell fractions from the high and low end of distributions, and measuring volume electronically to determine which sorting strategy yielded the best separated volume distributions. Since we found that different optical measurements were optimal for different cell lines, we suggest that following this procedure will enable other investigators to optimize their own cell sorters for volume-based separation of the cell types with which they work.

## Introduction

Clarifying relationships between cell cycle and cell growth is essential to understanding both normal and abnormal cell size, tissue architecture and organogenesis [1]. Recent studies in bacteria and yeasts provide evidence for mechanisms that regulate cell size [2], [3]; a molecular basis for doing this in fission yeasts has been proposed [4], [5]. In multicellular organisms, external factors may affect and regulate growth, proliferation and size; however, others' work on various animal cell types [6] and our recent studies of proliferating lymphoblastoid cells [7] suggest that mammalian cells must possess an cell-autonomous size regulation mechanism.

At present, the molecular nature of such a mechanism in animal cells is completely unknown, and its elucidation at genomic, proteomic and biochemical levels would be facilitated by use of a rapid method of separating large subpopulations of cells based on precise size measurements. In principle, it should be possible to use flow cytometric cell sorting [8], a technique now available in many laboratories, for this purpose.

The closest approximation to a “gold standard” for cell size determination employs electronic measurement (the Coulter principle). Since cells are poor conductors of electricity, the passage of a cell through a saline-filled orifice will transiently increase the impedance of the orifice, in proportion to the volume of saline displaced, and the Coulter volume measurement thus obtained is relatively independent of the shape of the cell. Although the now-standard method of sorting by droplet charging and deflection was first implemented in an apparatus that measured cell volume by impedance [9], the commercial cell sorters now available make only optical measurements. Which of these provides the most precise indicator of cell size may vary with cell type and also with the optical and electronic characteristics of the sorter. The procedure we describe here makes it possible to determine which optical measurement parameter(s) on a sorter is or are best for measuring the size of a given cell type, and what level of precision in size measurement can be attained, by making Coulter volume measurements of sorted cell fractions.

Modern cell sorters typically measure light scattered at small and large angles to an illuminating laser beam. The intensity of light scattered at small (0.5–2 degree) angles (forward scatter, abbreviated as FALS or FSC) is, according to Mie theory, determined to a substantial extent by the size of the scattering particles. Although FSC measurements, specifically the integral ("area") of a FSC pulse (FSC-A) are commonly described (e.g., on the web sites of institutional sorting facilities) as indicative of cell size, numerous publications, including textbooks on flow cytometry [8], [10], [11], [12] stress that such measurements are also influenced by the refractive index difference between particles and fluid, by absorbing substances inside cells or particles, and by the optical design of the FSC measurement system. In fact, FSC intensity has been demonstrated to increase monotonically with particle size in some instruments and not to do so in others [8], [13], [14]. The intensity of light scattered at large (15 to 135 degrees) angles (side scatter, abbreviated as SSC) is demonstrably affected by the composition or complexity of the cell. Any internal and surface irregularities, including cytoplasmic granules, vesicles, and other organelles and membrane roughness will typically contribute to SSC signals. SSC intensity also, however, increases with particle size, other things being equal, and has been shown to be well correlated with the intensity of fluorescence measured from dyes that stain most or all of the total protein in fixed cells from both pro- and eukaryotes [8], [15]. SSC has not been commonly used as a measure of cell size in flow cytometers, but has in apparatus designed for analysis of other particulates in air and liquids.

Many flow cytometers and sorters now in use focus an illuminating laser beam to an elliptical spot at the intersection of the beam and sample stream, with an axial dimension (beam height) smaller than a typical cell diameter oriented in the direction of sample flow. The durations ("widths") of the scatter and fluorescence pulses generated by cells' passage through such a tightly focused beam are therefore longer for cells of larger diameter, provided cell diameter is larger than beam height, and are described as representing the time of flight ("TOF") of the cell through the beam. The integral ("area") of a pulse provides an indication of the total amount of scattering or fluorescing material in a cell; the maximum intensity value ("peak" or "height") of a pulse provides an approximate measure of the highest concentration of scattering or fluorescing material. In older flow cytometers, pulse widths, heights, and areas were measured directly using digitally controlled analog electronics. In many newer ones, signal streams from the detectors are digitized at much higher data rates, allowing 16 or more samples of each pulse to be collected. The pulse height is equal to the maximum value among these samples; the area (integral) is computed from their sum, and a measure of pulse width is derived from the quotient of the area and the height. Pulse width (typically of an FSC signal) is most often used to discriminate cell doublets from single cells, but it has long been recognized as providing size information (see the detailed discussion in [16].

Although the multiparameter fluorescence measurement capabilities of flow cytometers make it possible to quantify a dozen or more intrinsically fluorescent cell constituents and/or fluorescently labeled macromolecules in or on a single cell, using excitation sources and detectors covering a broad range of wavelengths, fluorescence measurements have not been extensively evaluated as indicators of cell size. In addition to examining forward and side scatter pulse area (FSC-A and SSC-A) and forward scatter pulse width (FSC-W) in this regard, we also elected to evaluate cellular autofluorescence in respect to cell size, as this characteristic, like scatter characteristics, can be measured without the addition of a reagent to the cell sample, eliminating the possibility that such a manipulation might affect volume.

We measured scatter and autofluorescence parameters in several different cell types, in a cell sorter, sorting cells from the low and high ends of one- and two-dimensional distributions in each case. We then measured the volumes of cells from the sorted fractions in a Coulter counter, determining for each cell type which single parameters and two-parameter combinations provided the most widely separated volume distributions.

## Results and Discussion

### Different scatter measurements approximate cell size to different extents

**Figure 1:**
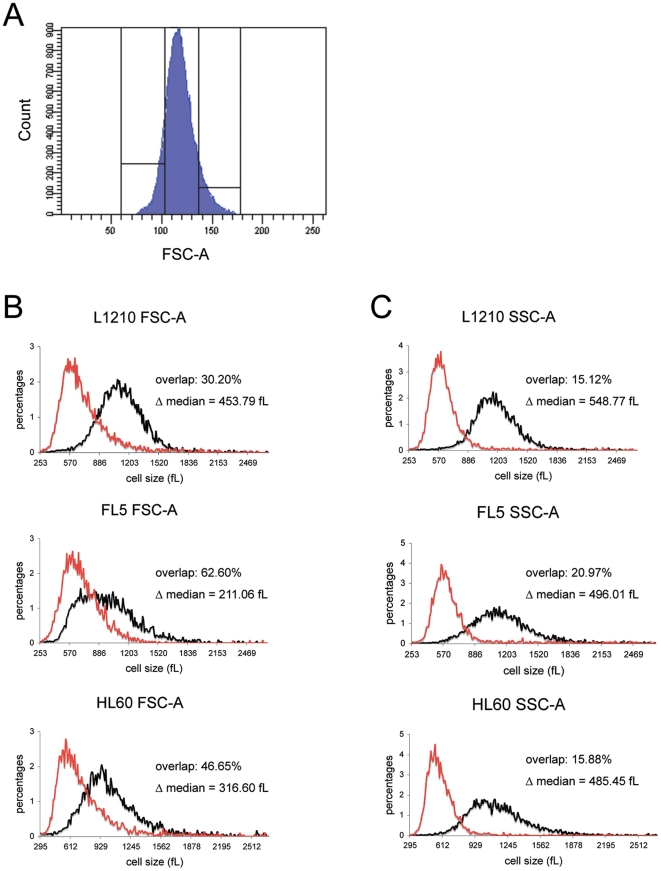
Cell size is better approximated by SSC-A rather than FSC-A. (**A**) Sort gates depicting the generalized sort scheme whereby the upper and lower 10% of the intensity distribution for the parameter of interest (FSC-A is shown in the figure) were sorted on a FACSAria. (**B**) L1210 (upper panels), and FL5.12 (middle panels) mouse cells, and HL60 human cells (lower panels) were sorted by FSC-A (left panels) and SSC-A (right panels) using the gating scheme shown in panel A. Volume distributions measured on a Coulter Counter are shown for cells isolated from the lower (red) and upper (black) sort gates. The overall quality of the size separation was estimated by two measures, the percent overlap and the difference in femtoliters (fL) between medians (Δ median) of the size distributions of the two sorted populations.

**Figure 2:**
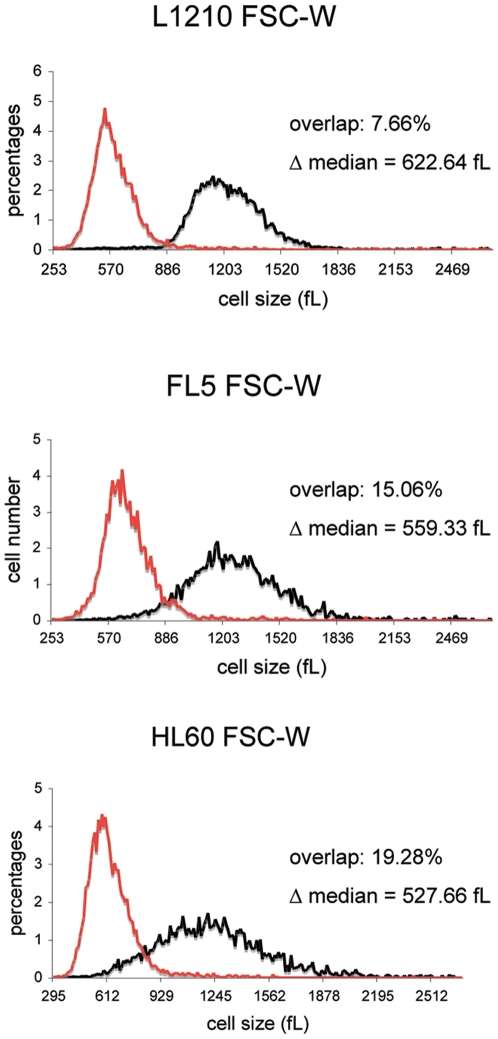
The signal width improves size separation by FSC. L1210 (upper), FL5.12 (middle), and HL60 (lower) cells were sorted by FSC-W. Size distributions, percent overlap and Δ median, were measured and calculated as in Figure 1.

To evaluate the relationship between light scatter parameters and cell size in mammalian cells, we set sort gates whereby the upper and lower 10% of the intensity distribution for different scatter parameters were sorted (the gating windows for FSC-A are shown in Figure 1A). Volume measurements of sorted cell fractions were subsequently made on a Coulter Counter. We used the calculated percent overlap and the difference in median values (Δ median) between the measured volume distributions of the “small” and “large” sorted fractions to describe the quality of the separation based on various surrogate parameters. The same protocol was applied to L1210 mouse lymphoblasts, FL5.12 mouse pro-B lymphocytes and human HL60 promyelocytic leukemia cells in order to elucidate possible differences among cell types with respect to which parameter(s) provided the best indication of size (Figures 1B, 1C, and 2).

A gating strategy similar to that used for FSC-A was applied to the SSC-A parameter (Figure 1C). For all cell types examined, a 2- to 3-fold decrease in size overlap was found when sorting was based on SSC-A rather than FSC-A. Moreover, the Δ median between the two size distributions was significantly higher (>2 fold for FL5.12 cells) for the SSC-A based separation (Figures 1B and 1C). Results from three independent experiments for the three cell types are summarized in Table 1.

**Table 1:** Quality of size-based separation based on various surrogate parameters.

	L1210			FL5			HL60		
	FSC	SSC	450/50 mm	FSC	SSC	450/50 mm	FSC	SSC	450/50 mm
**Area**	**32.69** **SD: 3.89**411.58SD: 55.84	**15.83** **SD: 0.99**527.66SD: 21.10	**22.67** **SD: 0.47**499.52SD: 24.37	**59.58** **SD: 2.81**239.20SD: 24.37	**21.69** **SD: 3.97**488.97SD: 21.96	**23.13** **SD: 3.90**474.89SD: 21.10	**41.97** **SD: 3.80**362.33SD: 42.64	**16.03** **SD: 0.52**513.59SD: 26.55	**8.64** **SD: 3.42**587.46SD: 12.18
**Width**	**8.54** **SD: 0.77**608.57SD: 16.12	**24.10** **SD: 2.94**467.86SD: 12.18	**38.09** **SD: 1.60**372.88SD: 16.12	**15.33** **SD: 6.03**576.91SD: 49.87	**30.24** **SD: 3.86**467.86SD: 24.37	**48.63** **SD: 2.45**344.74SD: 16.12	**18.85** **SD: 0.47**538.21SD: 10.55	**29.93** **SD: 1.63**411.58SD: 21.10	**37.77** **SD: 1.22**369.36SD: 10.55
**Height**	**56.47** **SD: 5.76**235.69SD: 49.87	**28.14** **SD: 1.41**411.58SD: 10.55	**87.87** **SD: 6.12**408.06SD: 87.87	**78.86** **SD: 0.16**119.60SD: 6.09	**38.20** **SD: 3.53**348.25SD: 10.55	**48.52** **SD: 3.79**299.01SD: 21.96	**76.78** **SD: 3.80**87.94SD: 37.06	**33.61** **SD: 1.25**355.29SD: 21.96	**20.37** **SD: 2.09**496.00SD: 73.87

Percent overlap (bold) and Δ median (fL) for separation of L1210 (Cols. 1–3), FL5.12 (Cols. 4–6), and HL60 (Cols. 7–9) cells using FSC (Cols. 1,4,7), SSC (Cols. 2,5,8), and 450 nm autofluorescence (Cols. 3,6,9). Parameter area data are in Row 1, width data in Row 2, and height data in Row 3. Each cell of the table contains data from 3 samples (mean values and SD are depicted).

We next investigated the relationship between the height and width of both FSC and SSC, and cell size. As shown in Table 1, sorting based on either FSC-H or SSC-H significantly reduced the quality of the size separation for all cell types, as did utilizing SSC-W as the gating parameter. Sorting based on FSC-W (Figure 2) clearly improved the size separation for both L1210 and FL5.12 (by >15%), with percent overlap reaching the lowest values of 8.5% and 15% respectively, but only minimally improved on the size separation achieved by SSC-A in HL60 cells.

### The cell's autofluorescence can indicate its size

Due to intrinsic optical properties of intracellular materials, illumination of the cell will result in autofluorescence. The major sources of autofluorescence emission at visible wavelengths are small molecules, notably NADPH (the reduced form of nicotinamide adenine dinucleotide phosphate) and flavin coenzymes [8]. The spectral range and the intensity of autofluorescence are expected to be different for different cell types or for cells at different growth conditions. However, for a cell line at uniform growth conditions, the fraction of intracellular fluorescent components (in mass), out of the total substances of the cell, may not vary much. If this is the case, the cell's autofluorescence would be expected to correlate with its mass, and therefore with its size.

**Figure 3:**
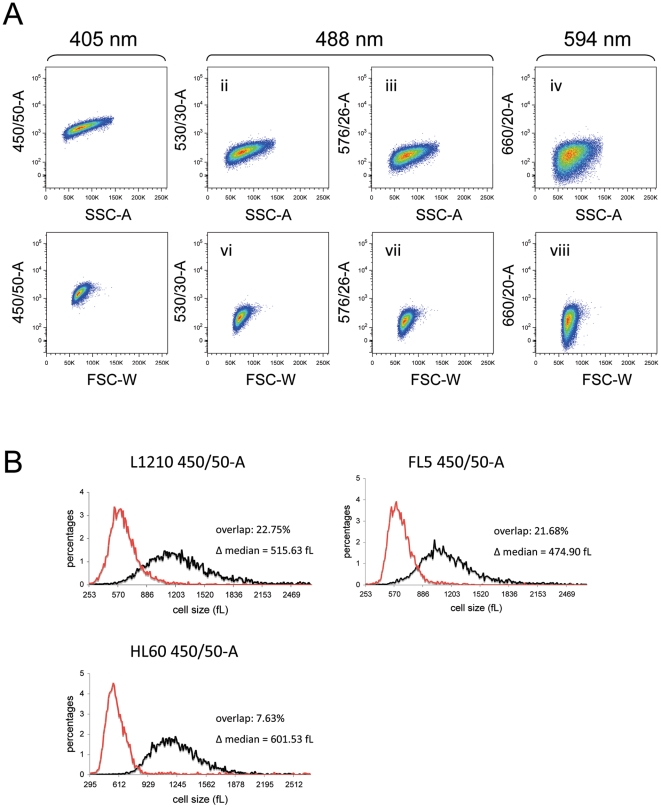
The cell's autofluorescence can indicate its size. (A) Bivariate plots of L1210 autofluorescence signal area versus SSC-A (i-iv) or FSC-W generated on FACSAria (v-viii). Autofluorescence was elicited utilizing the 3 indicated excitation wavelengths, and subsequently measured at the depicted bandwidths. Linear correlations between cellular autofluorescence and light scatter are summarized in Table 2. (B) L1210 (upper), FL5.12 (middle) and HL60 (lower) cells were sorted by autofluorescence intensity, elicited by 405 nm wavelength excitation and measured at 425-475 nm (450/50-A). Size distributions, percent overlap, and Δ median were measured and calculated as in Figure 1B.

Using SSC-A and FSC-W as proven references for cell size (Figures 1 and 2), we analyzed the correlation between these two parameters and autofluorescence (signal Area only) in L1210, FL5.12 and HL60 cells. We examined violet (405 nm)- excited blue (450 nm, 50 nm bandwidth) fluorescence, blue (488 nm)-excited green (530 nm, 30 nm bandwidth) and yellow (576 nm, 26 nm bandwidth) fluorescence, and yellow (594 nm)-excited red (660 nm, 20 nm bandwidth) fluorescence. Figure 3A and Table 2 show the correlations between SSC-A (plots i-iv) or FSC-W (plots v-viii) and autofluorescence. Correlation with both scatter parameters reached the highest R^2^ value when autofluorescence was excited by the 405 nm laser and measured at 450 nm (plots i and v) or at 525 nm (data not shown).

**Table 2:** Correlation between light scatter parameters and autofluorescence.

Excitation	Bandwidth (nm)	R^2^ SSC-A	R^2^ FSC-W
405 nm	425–475 nm	0.68	0.48
488 nm	515–545 nm	0.50	0.39
488 nm	563–589 nm	0.40	0.31
594 nm	650–670 nm	0.14	0.13

We sorted cells with the 10% lowest and 10% highest 405 → 450 nm autofluorescence intensity, based on the area, the height, and the width of the signal, and measured the Coulter volume distributions of the sorted populations (Figure 3B). When sorting was based on the area of the signal (450/50-A) the quality of the size separation (estimated by the Δ median and the percent overlap between the two size distributions) was between 1.7-fold and 8-fold better than the separation achieved by the signal height (450/50-H) or the signal width (450/50-W) for all three cell types (Table 1).

In L1210 and FL5.12 cells, separation was considerably better than that achieved by FSC-A and only slightly inferior to FSC-W and SSC-A based separation. For HL60 cells, 450/50-A-based sorting provided the best separation achieved in this study, with the lowest overlap (8.64%) and the highest Δ median value (587 fL) (Table 1, also see Figure 3B).

These data indicate that 405 → 450 nm autofluorescence can serve as a surrogate for cell size measurement, at least for some mammalian cells, but that which parameter(s) is/are optimal for any given cell type are best determined empirically.

### Flow cytometric cell size estimation my be improved by combining optical parameters

FSC-W, SSC-A and 450/50-A autofluorescence, which all correlate well with the actual size of cells, reflect very different biophysical and biochemical characteristics, and are therefore likely to approximate size for different reasons. We therefore investigated whether a combination of optical cytometric parameters could provide more accurate information about cell size, and better separation, than a single parameter. Because the interface of our FACSAria sorter allows the user to set thresholds visually (through gates) but not numerically, we applied a sequential Boolean gating strategy to sort cells using a combination of parameters. Since FSC-W provided the maximum size separation on L1210 cells (Table 1), we used L1210 for these experiments and set FSC-W as the first gating parameter.

**Figure 4:**
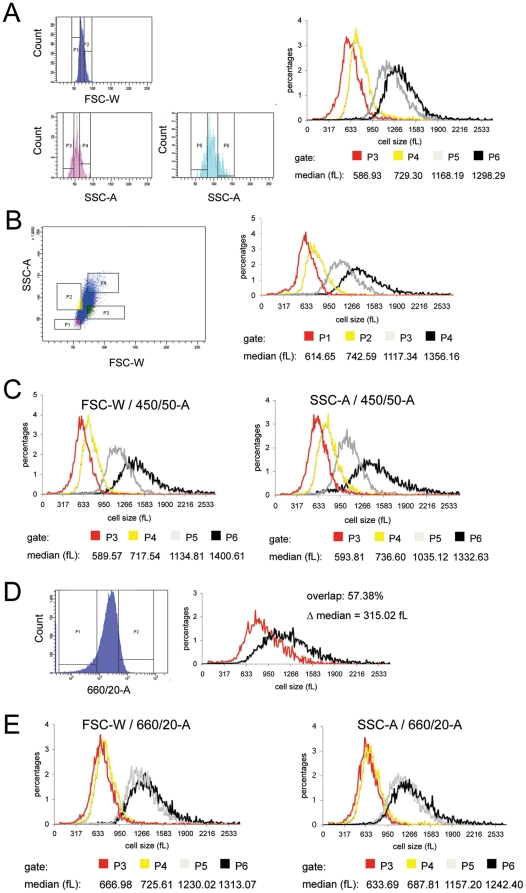
Cell size is better approximated by a combination of optical parameters. (**A**) L1210 cells were sorted utilizing a sequential boolean gating strategy. Gates P1 and P2 (see upper left panel) included the upper and lower 20% of the FSC-W distribution. The subsequent sort gates P3, P4 and P5, P6 (see lower left panels) were based on the upper and lower 20% of the SSC-A distributions of the “low” (P1 gate) and the “high” (P2 gate) FSC-W populations. The size distributions of the four sorted populations were analyzed on the Coulter Counter. Depicted are the median size values of each population. (**B**) Sort gate operations performed in (A) were repeated utilizing a bivariate gating strategy resulting in 4 gates with a final 4% of the total population in each (left panel). The size distribution of the four sorted populations (right panel) was measured as in (A). (**C**) A sequential boolean gating strategy, similar to that used to set sort gates in (A), was utilized for FSC-W and 450/50-A autofluorescence parameters (left panel) and for SSC-A and 450/50-A autofluorescence parameters (right panel). The size distribution and the median size values are depicted. (**D**) L1210 cells were sorted by 594 nm-excited 660/20-A autofluorescence (660/20-A; APC-A). The upper and lower 10% of the distribution were sorted for size measurement (see Figure 1 for details). (**E**) Experiment described in 4C repeated for FSC-W and 660/20-A autofluorescence parameters (left panel) and for SSC-A and 660/20-A autofluorescence parameters (right panel).

Gates P1 and P2 (Figure 4A, upper left panel) included the upper and lower 20% of the FSC-W distribution. The subsequent sort gates were based on the upper (P3, P4) and the lower (P5, P6) 20% of the SSC-A distributions of the “low” (P1 gate) and the “high” (P2 gate) FSC-W populations (Figure 4A lower left panels). In theory, if SSC-A and FSC-W approximate size on similar bases, cells selected by gates P3 and P4 should have the same size distribution, as should cells selected by gates P5 and P6. If, however, SSC-A provides additional information about cell size, we would expect to see two-step separation resulting in four different size distributions. Results (Figure 4A right) show that combining FSC-W and SSC-A in fact does generate four distinguishable distributions.

An additional gating strategy was used to rule out a gating artifact. We created a bivariate dot-plot with FSC-W and SSC-A. First we defined two gates (P1 and P2) capturing 20% of the cells with the lowest FSC-W intensity and two additional gates (P3 and P4) that captured 20% of the populations with the highest FSC-W intensity. Than we shifted the gates on the SSC-A axis to define four sorting gates that each capture 4% of the total population (Figure 4B left) (4% represents the equivalent of sequential gating of 20% out of 20%). These gates represent the 20% lowest FSC-W/20% lowest SSC-A (P1), 20% lowest FSC-W/20% highest SSC-A (P2), 20% highest FSC-W/20% lowest SSC-A (P3) and 20% highest FSC-W/20% highest SSC-A (P4), recapitulating the previous experiment. This gating strategy also provided four distinct size distributions (Figure 4B right), similar to those shown in Figure 4A.

We next combined FSC-W and 450/50-A parameters, and SSC-A and 450/50-A parameters, as we had the FSC-W and SSC-A parameters. In both experiments, we observed two-step separation of sizes resulting in four distinct populations, with sequential Boolean and single bivariate gating producing similar results (Figure 4C and Figures S1 and S2).

As a control for the gating strategies just described, we combined FSC-W or SSC-A with 594 nm-excited autofluorescence at 650-670 nm (660/20-A or APC-A), a parameter we had shown to correlate poorly with both SSC-A and FSC-W (Figure 3A and Table 2). 660/20-A is indeed ineffective for size separation (Figure 4D) and therefore is unlikely to substantially influence the separation achieved by FSC-W or SSC-A. Results as shown in Figure 4E, left panel, indicate that the cells with the upper and lower 20% 660/20-A intensity gated from either the 20% “low” FSC-W or the 20% “high” FSC-W populations (P3, P4 and P5, P6) greatly overlap in their sizes. Equivalent results were obtained when 660/20-A was combined with SSC-A (Figure 4E, right panel, P3, P4 and P5, P6), i.e, rather than showing a two-step separation resulting in four clearly resolved populations, the addition of the 660/20-A autofluorescence parameter did not significantly enhance the separation achieved by the scatter gating parameter alone (see also Figures S3 and S4).

## Discussion

Here we show that SSC, cell autofluorescence and FSC-W are good proxies for cell size and can be used for size-based sorting in flow cytometry. We note, however, that before beginning experiments with cells, we measured FSC and SSC area, height, and width of four sizes of polystyrene beads (3, 5, 7, and 10 µm diameter) on our cell sorter and verified that modal intensities of the peaks of all distributions increased monotonically with bead diameter (data not shown). This monotonic relationship does not exist for some FSC measurements made in other flow cytometers, and we would suggest that the validity of FSC-W be verified empirically for measurements made in such apparatus. We also note that FSC-A, although ordained by "common knowledge" as a preferable size measurement parameter, approximated cell size less well than other parameters, even in our instrument, in which a monotonic relationship with bead size was present.

Although old literature has linked SSC to total protein content of cells as measured using fluorescent dyes, and the latter parameter is accepted to be the primary determinant of cells' interferometric dry mass [17], flow cytometric measurements of SSC have not been widely used to approximate cell size. In situations compatible with the addition of the necessary reagent, it could be worthwhile to reexamine the use of total protein staining as a size indicator.

Our demonstration of the use of violet (405 nm)-excited blue (450 nm) autofluorescence as a surrogate for cell volume naturally raises the question of why this parameter should be so well correlated with size; it is not within the scope of the present work to provide an answer. What we emphasize here is, first, that none of the four optical parameters (FSC-A, FSC-W, SSC-A, and 450/50-A) we investigated in four cell types provided the best indication of cell volume for all cell types and, second, that a combination of light scattering and fluorescence parameters was likely to approximate cell size of any given cell type better than any individual optical parameter.

It would, obviously, be preferable if size-based cell separation could be made simpler. Although we have recently made use of a highly accurate but technologically demanding electromechanical technique for determining the wet weights of individual growing cells [3], we are not optimistic that this can be adapted to separation on anything like the scale possible using cell sorters. Conventional sorters that combined Coulter volume measurements and fluorescence measurements were built in laboratories in the 1970s; increased interest in cell size and its biological role might stimulate commercial production of such devices.

There is also the possibility that highly accurate and precise optical interferometric techniques for cell dry mass measurement, known for decades but greatly improved of late [17], [18] will be incorporated into the toolboxes of the current generation of sorters. In this context, dry mass-based sorting of X- and Y-chromosome-bearing sperm has already been attempted [19], and the demand for improvements of this technology in animal husbandry may, appropriately, provide cross-fertilization that will benefit cell and systems biologists.

In the real world in which we live and work, however, cell sorters cost hundreds of thousands of dollars, and modifying them substantially costs tens of thousands of dollars. Most laboratories which now have cell sorters also have impedance-based cell counters or can acquire them for no more than a few thousand dollars, allowing reasonably good volume-based cell separation to be done as we have described with existing resources.

### Conclusions

Although four optical scatter and fluorescence parameters (FSC-A, FSC-W, SSC-A, and 450/50-A) measurable in existing commercial cell sorters provide estimates of cell volume, an examination of electronic (Coulter) cell volume distributions in fractions of four mammalian cell lines sorted from the high and low ends of optical parameter distributions showed that no one parameter provided the best volume estimate for all four lines, and that combinations of parameters could estimate volume better than single parameters. At the present state of the art, similar empirical analyses can provide the best approximation to volume-based sorting that can be achieved with existing equipment.

## Materials and Methods

### Cell Culture

Murine L1210 lymphoblasts cells were cultured in Leibovitz's L-15 media (Invitrogen) supplemented with 10% FBS (Invitrogen), 1.8 g/L D-(+)-glucose solution (Sigma-Aldrich) and 1% 100× penicillin-streptomycin solution (Gemini). Murine pro-B lymphocytes FL5.12 stably transfected with BCL2 cells were kindly provided by Craig Thompson. Cells were grown in RPMI media (Invitogen) supplemented with 10% FBS (Invitrogen), 25 mM HEPES, 1% 100× penicillin-streptomycin solution (Gemini), 0.5 mg/ml Geneticin[TM] (Invitrogen), 55 mM 2-Mercaptoethanol and 3 ng/ml mouse recombinant Interleukin-3 (BD Biosciences, # 354058). Human HL-60 promyelocytic leukemia cells (ATCC #CCL-240) were cultured in RPMI media, supplemented with 10% FBS (HyClone) and 1% 100× penicillin-streptomycin solution (Gemini).

### Cell Sorting and size measurements

L1210, FL5 and HL60 cells from exponentially growing cultures were centrifuged at 200 g (5 minutes) and resuspended in phenol red-free L-15 (L1210) or RPMI (FL5, HL60) media supplemented with 2% FBS, at a concentration between 15–20×10^6^ cells/ml. The cell suspensions were filtered through BD Falcon Cell-Strainer Caps (352235) and then sorted on a BD FACSAria IIu at 20 psi using a 100 µM nozzle at a flow rate of “1.0”. Cell aggregates were removed from the analysis using a sequential gating strategy relying first on FSC height versus width followed by SSC height versus width parameters, as recommended by BD (see BD FACService TECHNOTES, Customer Focused Solutions, Vol. 9 No. 4 October, 2004). Multiple sized microspheres were purchased from Spherotech (cat. # PPS-6K) and analyzed on the FACSAria IIu.

For initial size separation sorting we utilized a single parameter histogram with gates isolating the lower and upper 10% of the intensity distribution of the chosen parameter, unless otherwise indicated. Sequential boolean gating strategies are described in detail in the text (See Figure 4). Light scatter parameters were measured using the 488 nm laser. Excitation/emission parameters are described in the text for each experiment. FACS data was prepared for presentation using FlowJo v. 8.1. The size distribution of the sorted cells was determined using the Z2 Coulter Counter and Multisizer III software (Beckman Coulter). Microsoft Excel was used for data analysis.

## Supporting Information

Figure S1 (**A**) L1210 cells were sorted utilizing a sequential Boolean gating strategy. Gates P1 and P2 (see upper panel) included the upper and lower 20% of the FSC-W distribution. The subsequent sort gates P3, P4 and P5, P6 (see lower panels) were based on the upper and lower 20% of the 405-excited 450/50-A distributions of the “low” (P1 gate) and the “high” (P2 gate) FSC-W populations. (**B**) Same gating strategy used with SSC-A and the 405-excited 450/50-A parameters.
(TIF)

Figure S2 (**A**) Sort gate operations utilizing a bivariate gating strategy resulting in 4 gates with a final 4% of the total population in each (left panel). Same gating strategy used also with SSC-A and the 405-excited 450/50-A parameters (**B**). The size distribution of the four sorted populations was measured using Coulter Counter (**A** and **B** right panels). Depicted are the median size values in femtoliters (fL) of each population.
(TIF)

Figure S3 (**A and B**) L1210 cells were sorted utilizing a sequential Boolean gating strategy. Gates P1 and P2 included the upper and lower 20% of the FSC-W (A, upper panel) or the SSC-A (B, upper panel) distributions. The subsequent sort gates P3, P4 and P5, P6 (see A and B lower panels) were based on the upper and lower 20% of the 594nm-excited 660/20-A distributions of the “low” (P1 gate) and the “high” (P2 gate) FSC-W (A) or SSC-A (B) populations.
(TIF)

Figure S4 (**A**) Sort gate operations utilizing a bivariate gating strategy resulting in 4 gates with a final 4% of the total population in each (left panels). Same approach was used also for SSC-A and 594nm-excited 660/20-A parameters (**B**). The size distribution of the four sorted populations was measured using Coulter Counter (**A** and **B** right panels). Depicted are the median size values of each population.
(TIF)
