# Supplementary figures and images for: Optimizing Optical Flow Cytometry for Cell Volume-Based Sorting and Analysis
Source: PLoS One. 2011 Jan 20;6(1):e16053. doi: 10.1371/journal.pone.0016053 (PMC3024321; doi:10.1371/journal.pone.0016053)

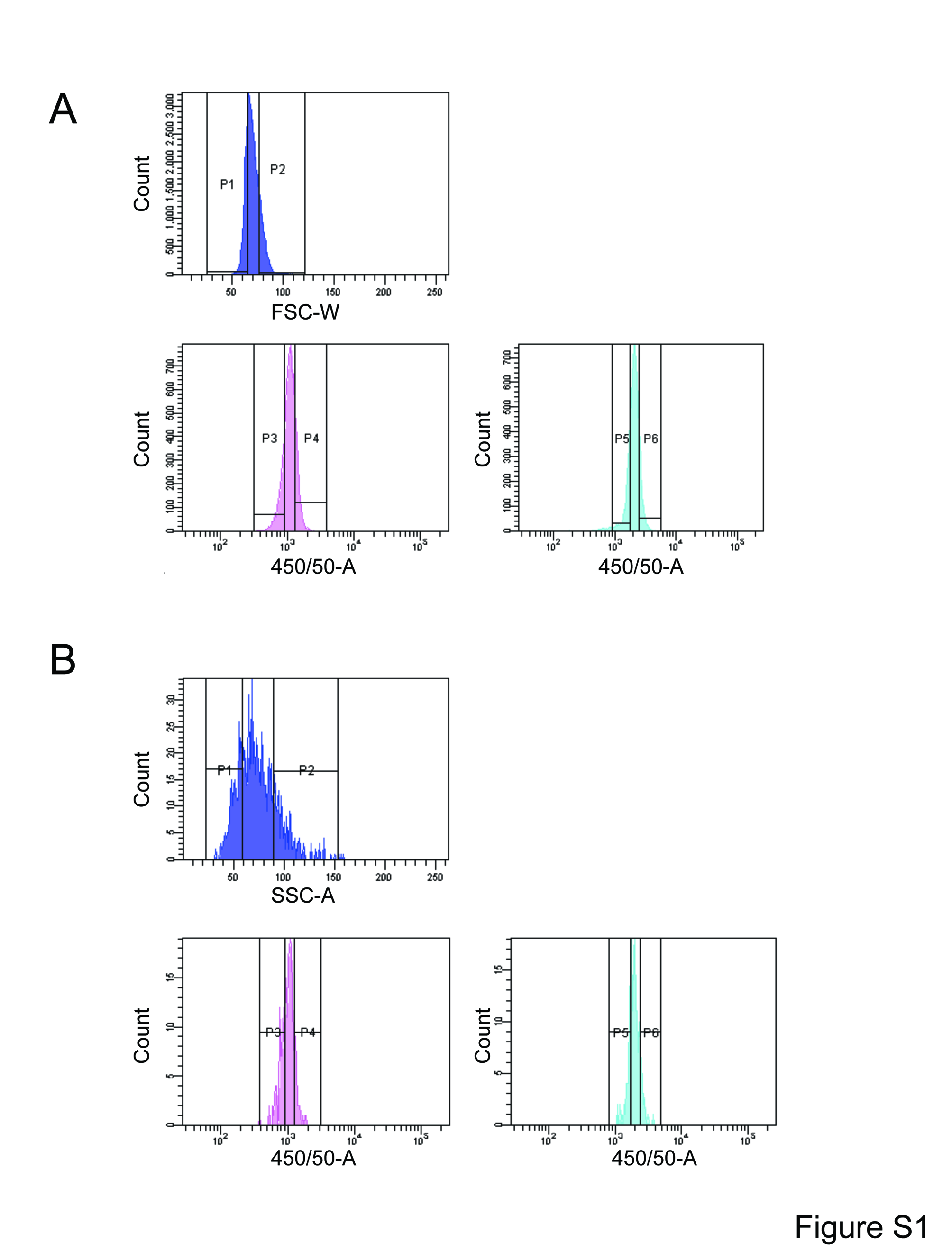

Supplement: Figure S1 — (A) L1210 cells were sorted utilizing a sequential Boolean gating strategy. Gates P1 and P2 (see upper panel) included the upper and lower 20% of the FSC-W distribution. The subsequent sort gates P3, P4 and P5, P6 (see lower panels) were based on the upper and lower 20% of the 405-excited 450/50-A distributions of the “low” (P1 gate) and the “high” (P2 gate) FSC-W populations. (B) Same gating strategy used with SSC-A and the 405-excited 450/50-A parameters. (TIF) [file pone.0016053.s001.tif]

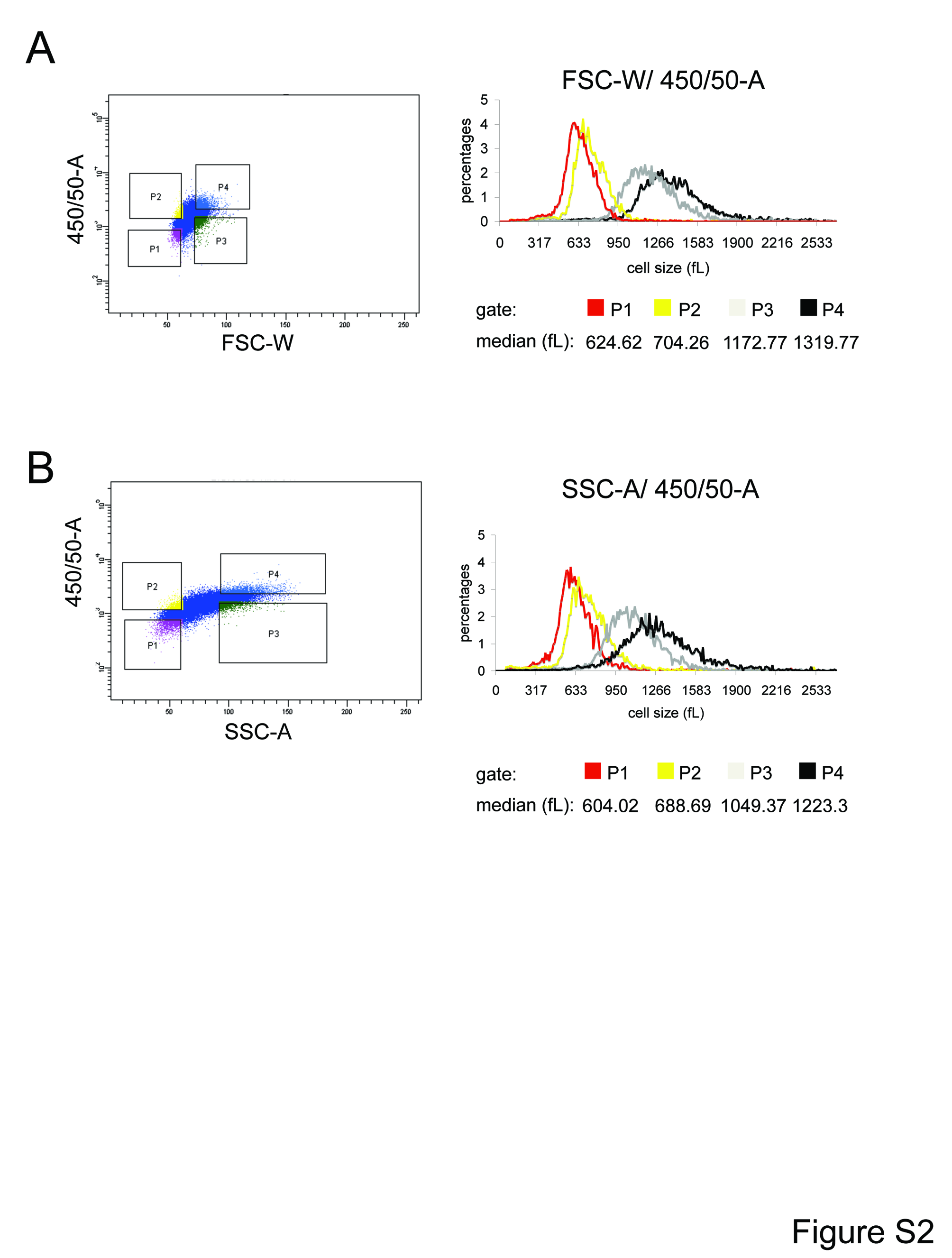

Supplement: Figure S2 — (A) Sort gate operations utilizing a bivariate gating strategy resulting in 4 gates with a final 4% of the total population in each (left panel). Same gating strategy used also with SSC-A and the 405-excited 450/50-A parameters (B). The size distribution of the four sorted populations was measured using Coulter Counter (A and B right panels). Depicted are the median size values in femtoliters (fL) of each population. (TIF) [file pone.0016053.s002.tif]

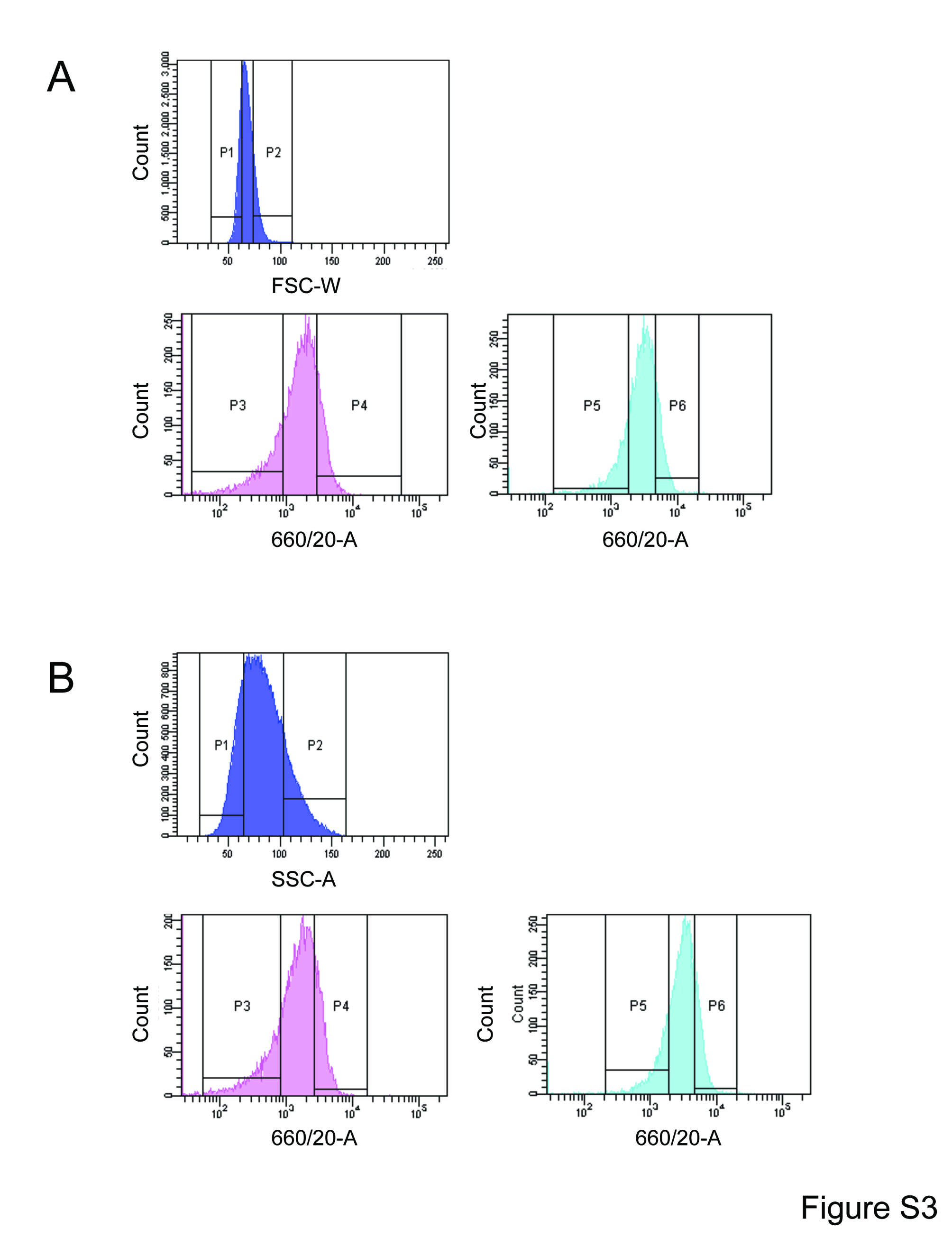

Supplement: Figure S3 — (A and B) L1210 cells were sorted utilizing a sequential Boolean gating strategy. Gates P1 and P2 included the upper and lower 20% of the FSC-W (A, upper panel) or the SSC-A (B, upper panel) distributions. The subsequent sort gates P3, P4 and P5, P6 (see A and B lower panels) were based on the upper and lower 20% of the 594nm-excited 660/20-A distributions of the “low” (P1 gate) and the “high” (P2 gate) FSC-W (A) or SSC-A (B) populations. (TIF) [file pone.0016053.s003.tif]

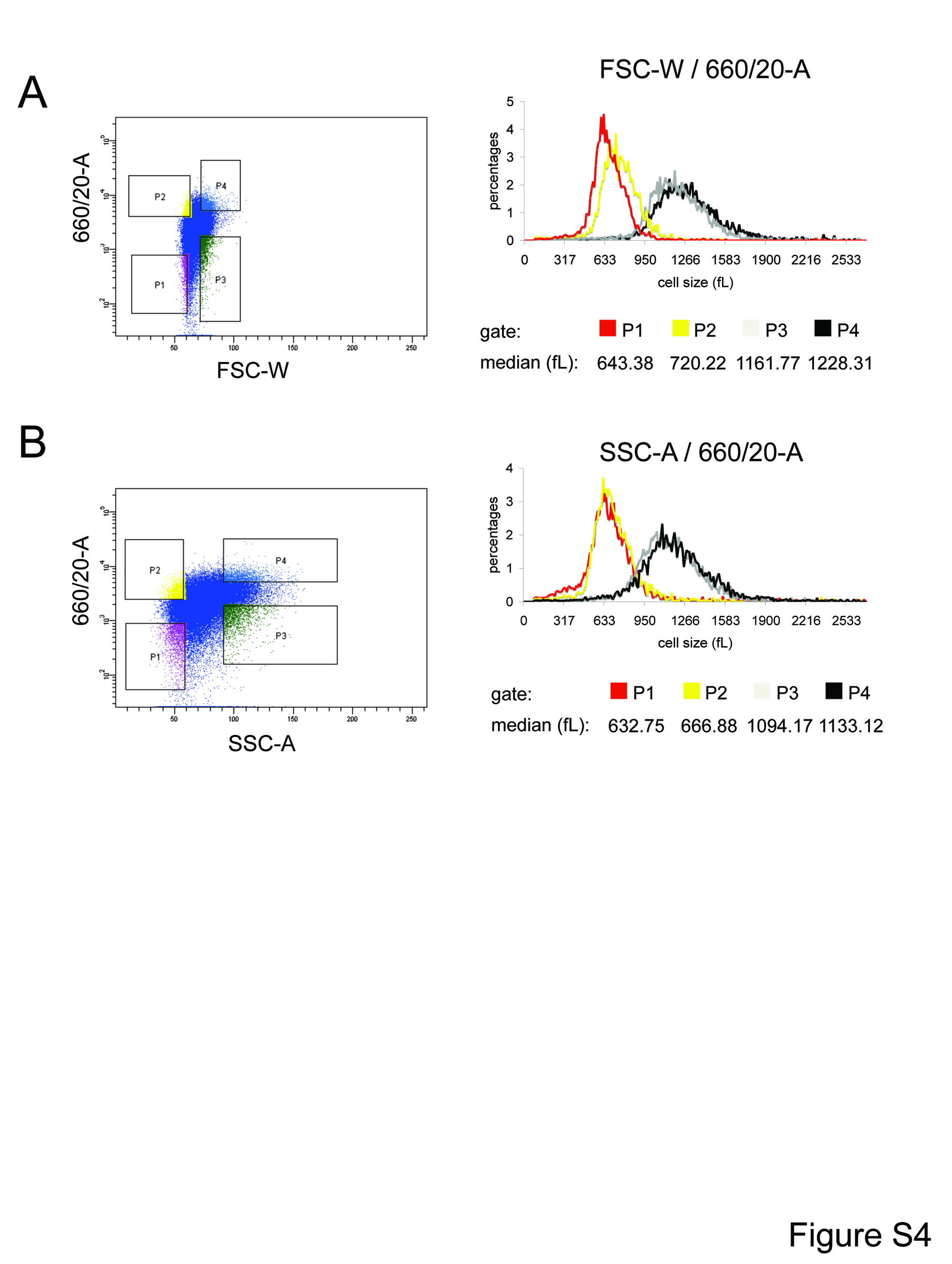

Supplement: Figure S4 — (A) Sort gate operations utilizing a bivariate gating strategy resulting in 4 gates with a final 4% of the total population in each (left panels). Same approach was used also for SSC-A and 594nm-excited 660/20-A parameters (B). The size distribution of the four sorted populations was measured using Coulter Counter (A and B right panels). Depicted are the median size values of each population. (TIF) [file pone.0016053.s004.tif]
